# Inferring Interaction Networks From Multi-Omics Data

**DOI:** 10.3389/fgene.2019.00535

**Published:** 2019-06-12

**Authors:** Johann S. Hawe, Fabian J. Theis, Matthias Heinig

**Affiliations:** ^1^Institute of Computational Biology, HelmholtzZentrum München, Munich, Germany; ^2^Department of Informatics, Technische Universität München, Munich, Germany; ^3^Department of Mathematics, Technische Universität München, Munich, Germany

**Keywords:** systems biology, genomics, prior information, machine learning, personalized medicine, data integration, single cell, mixed data

## Abstract

A major goal in systems biology is a comprehensive description of the entirety of all complex interactions between different types of biomolecules—also referred to as the interactome—and how these interactions give rise to higher, cellular and organism level functions or diseases. Numerous efforts have been undertaken to define such interactomes experimentally, for example yeast-two-hybrid based protein-protein interaction networks or ChIP-seq based protein-DNA interactions for individual proteins. To complement these direct measurements, genome-scale quantitative multi-omics data (transcriptomics, proteomics, metabolomics, etc.) enable researchers to predict novel functional interactions between molecular species. Moreover, these data allow to distinguish relevant functional from non-functional interactions in specific biological contexts. However, integration of multi-omics data is not straight forward due to their heterogeneity. Numerous methods for the inference of interaction networks from homogeneous functional data exist, but with the advent of large-scale paired multi-omics data a new class of methods for inferring comprehensive networks across different molecular species began to emerge. Here we review state-of-the-art techniques for inferring the topology of interaction networks from functional multi-omics data, encompassing graphical models with multiple node types and quantitative-trait-loci (QTL) based approaches. In addition, we will discuss Bayesian aspects of network inference, which allow for leveraging already established biological information such as known protein-protein or protein-DNA interactions, to guide the inference process.

## 1. Introduction

Systems biology aims to model complex biological systems by employing a holistic view on all cellular processes (Ideker et al., 2001). At its heart lies the central dogma of biology (Crick, 1958), i.e., genes encoded in the DNA (genome) are transcribed to mRNAs (transcriptome) which are translated to proteins (proteome). Additionally, other omic layers like the methylome (DNA methylation at CpG dinucleotides) and the metabolome (abundance of metabolites) take part in maintaining biological systems through molecular interaction networks. These lay the foundation for cellular processes such as gene expression regulation and metabolism. The working hypothesis of systems biology is that understanding molecular interactions and the regulatory networks they form is crucial to understand system level properties such as diseases or other phenotypes (Ideker et al., 2001).

Therefore, a goal of systems biology is to establish *interactomes*: networks of interacting molecules of distinct cellular omic layers. We define an interactome as a network consisting of nodes representing individual molecules and connections between nodes (edges) which reflect (1) physical (direct) or (2) functional (indirect) interactions between molecules. To establish physical interactions, experimental assays systematically interrogating direct interactions between molecules can be applied. For example, a protein interactome based on *protein-protein* interactions (PPIs, e.g., protein complexes), can be determined by large-scale yeast-2-hybrid screens (Y2H) or affinity purification followed by mass-spectrometry (AP-MS) (Brueckner et al., 2009). Furthermore, genome-wide *protein-DNA* interactomes can be constructed by chromatin immunoprecipitation followed by next-generation sequencing (ChIP-seq) (Johnson et al., 2007) in order to identify for instance all sites in the genome, where a particular transcription factor (TF) binds. Similarly, *protein-RNA* interactions can be probed using cross-linking immunoprecipitation (CLIP-seq) (Licatalosi et al., 2008; Van Nostrand et al., 2016). In addition, DNA-DNA and RNA-RNA interactomes can be established using Chromosome Conformation Capture (Hi-C) (Belton et al., 2012) or RAP-RNA sequencing (Engreitz et al., 2014), respectively.

Indirect functional interactions can be established experimentally through synthetic genetic array (SGA) screens (genetic interactions) (Costanzo et al., 2010) or by computational approaches such as co-regulation (determined from ChIP-seq or co-expression analyses) or co-evolution (Marcotte et al., 1999; De Bodt et al., 2006). For instance, if two genes both are always active in one set of samples and inactive in another set, one might conclude that the two genes are functionally related, based on the principle of guilt by association. This hence would allow to infer a hitherto unknown function of one gene if the function of the other gene is known.

In contrast to experimental protocols enabling to assess global omics profiles in arbitrary cellular contexts with relative ease, physical interaction probing cannot easily be applied to a broad range of biological contexts due to non-physiological conditions (e.g., Y2H) or the limited scope of one-to-many interaction profiling (e.g., AP-MS). Similar to reference genome sequences which are frequently used to provide a coordinate system for the analysis of DNA related processes, context-independent “reference interactomes” can serve as scaffolds to complement cell type or condition (e.g., disease) dependent analyses and several resources aim to provide these for numerous organisms (Table 1). These data set the stage for identifying context specific functionally relevant interactions and novel analysis methodologies need to be developed to derive or complement interactomes using functional genomics data.

**Table 1:** Overview on selected resources for molecular interactions and omics datasets.

**Resource**	**Data type**	**Organisms**	**References**
STRING	P-P^a^	> 5000	Szklarczyk et al., 2015
BioGrid	P-P	> 60	Stark et al., 2006
inBio map	P-P	HS	Li et al., 2017
GWAS catalog	D-PH	HS	MacArthur et al., 2017
KEGG	multiple	> 5000	Kanehisa and Goto, 2000
APID	P-P	> 400	Alonso-Lopez et al., 2016
doRINA	P-R, miR-R	HS, MM, DM, CE	Blin et al., 2015
REMAP	P-D	HS	Chèneby et al., 2018
IntAct	P-P^b^	multiple	Orchard et al., 2014
Pathway Commons	multiple	multiple	Cerami et al., 2011
AGRIS	P-D	AT	Yilmaz et al., 2011
ENCODE	G, T, E	HS	The ENCODE Project Consortium, 2012
modENCODE	G, T, E	DM, CE	Celniker et al., 2009
GTEx	G, T	HS	Carithers et al., 2015
ROADMAP	E, T	HS	Roadmap Epigenomics Consortium, 2015
GEO	G, T, E	multiple	Edgar et al., 2002; Barrett et al., 2013
ARCHS4	T	HS, MM	Lachmann et al., 2018
The Human Protein Atlas	T, P	HS	Thul et al., 2017
MetaboLights	M	multiple	Haug et al., 2013
TCGA	G, T, E	HS	Weinstein et al., 2013

*Data type column depicts either the type of interactions (e.g., protein-protein interaction, P-P) or the type of omics data available in the data collection. Interactions: M, metabolite; P, protein; D, DNA; R, RNA; PH, phenotype; Organisms: HS, H. sapiens; AT, A. thaliana; MM, M. musculus; DM, D. melanogaster; CE, C. elegans; Omics: G, genomic; E,epigenomic; T,transcriptomic*.

a *includes functional interactions*.

b *focus on P-P, but arbitrary interactions possible*.

Rich functional genomic data across large numbers of samples and across multiple omics layers per sample have been accumulated in several large scale projects, paving the way for a systematic integration of reference interactomes with context specific multi-omics data (see Box 1, resources listed in Table 1). These data allow researchers to link static interactomes to disease (e.g., *TCGA* in cancer) or tissue specific (e.g., *GTEx*) contexts and have already furthered our understanding of e.g., cancer mechanisms (Manatakis et al., 2018) or tissue specific gene regulation (Saha et al., 2017).

Box 1 **Glossary.**

**Table:** 

**Multi-omics data**	A dataset in which for each individual sample at least two different kinds of molecular information (such as genotype, gene expression, or DNA methylation information) is available.
**Partial correlation**	Measure of (conditional) dependence between (statistical) variables. Two variables are partially correlated, if they are still significantly correlated after the effect of all other variables in the dataset has been removed from the two target variables via linear regression. For multivariate normal distributions a partial correlation of zero is equivalent to conditional independence between two variables (Baba et al., 2004).
**Precision matrix**	In a Gaussian Graphical Model, where the *p* random variables represented in the nodes follow a multivariate Gaussian distribution, the precision matrix is the inverse of the covariance matrix. When normalized similarly as the correlation matrix, the entries in the *p* × *p* sized matrix correspond to the partial correlations between the respective variables.
**Regularization**	In a statistical model, the number of variables *p*, specifically the content of the variable coefficient vector β_1…*p*_, determines its complexity. Regularization can be applied to penalize model complexity. For example, *L*_1_ regularization employed in the LASSO pushes variable coefficients toward zero, effectively performing variable selection and reducing model complexity.
**Causal networks**	Causal networks (also Bayesian networks) are directed acyclic graphs and establish directed dependencies between individual nodes, i.e., all edges between nodes are effectively arrows representing a direction of effect. For example, in a causal co-expression network it could be deduced that the expression of a gene changes as a result of a change in another gene, while in an undirected network this would be reflected as a mere correlation.

They can further help in interpreting non-coding DNA sequence variants (single nucleotide polymorphisms, SNPs) from genome-wide association studies (GWAS). Integration of GWAS results with interaction data can pinpoint SNPs and their molecular targets causal to the respective GWAS phenotype (e.g., Hosp et al., 2015; Suhre et al., 2017). Additionally, databases like *GTEx* allow to interrogate tissue specific functional consequences of non-coding GWAS SNPs (Albert and Kruglyak, 2015; Aguet et al., 2017).

As protocols to measure functional genomics data get further developed, more possibilities to establish context specific interactomes arise. Single-cell nucleosome, methylation, and transcription sequencing (scNMT-seq) (Clark et al., 2018), for example, allows to generate multi-omics profiles of single cells. This, and single-cell experiments in general, open up promising new avenues for analyzing regulatory pathways in cellular systems: For instance, with single-cell data it is now possible to look at associations between variables in a more conventional statistical setting with at least as many or more samples (single cells) as measured variables, which is usually not the case in typical (bulk) omics studies (see section 2.2). Moreover, single-cell resolution further allows to extract dynamic properties of cellular systems on the basis of static snapshot data, making it possible to for example infer differentiation specific regulatory networks (Ocone et al., 2015). Single-cell data, however, come with their own challenges, for instance a high number of missing values due to low coverage per cell or dropout effects, and these have to be overcome in order to use them to their full potential.

**Figure 1:**
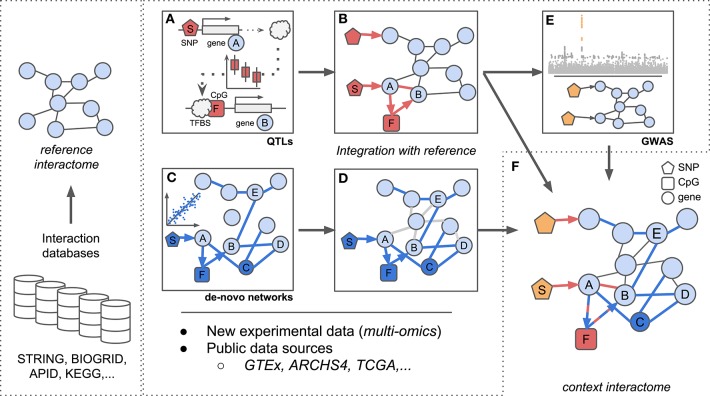
Scheme for integration of reference interactomes with multi-omics data and phenotypes (GWAS) to obtain context specific interactomes. **(A)** *trans*-eQTL allow to investigate e.g., TF binding mechanisms which can be complemented with additional regulatory information such as CpG methylation. **(B)** Established associations from **(A)** between SNP *S*, TF *A*, CpG *F*, and gene *B* complement reference interactomes. **(C)** Regulatory (possibly heterogeneous) networks are inferred from multi-omics data optionally using established biological knowledge as prior information. Integration of e.g., genotypes, expression, and methylation data allows to investigate regulatory dependencies between different omic layers. **(D)** Associations identified in **(C)** complement reference interactomes by adding new regulatory layers (SNP *S*, CpG *F*), novel genes (gene *C*) or new links between already existing genes (genes *B* and *E*) similar to (B). **(E)** Reference interactomes are annotated with SNPs associated with specific disease contexts from GWAS results. **(F)** The final context-specific interactome enables detailed investigation of disease related regulatory mechanisms across distinct omic layers.

Global interaction networks are important assets for systems biologists, yet their construction is not trivial for dynamic biological systems and novel methods need to be developed Here we present state-of-the-art methods for inferring interaction networks from multi-omics data. We will focus on two inference concepts which we term asynchronous and synchronous methods: asynchronous methods integrate multi-omics data in a step-by-step fashion, two omics at a time while synchronous methods incorporate all data concurrently (Figure 1). We will describe the inference of homogeneous and heterogeneous networks, i.e., networks consisting of a single or multiple node types, respectively, and further consider integration of prior biological knowledge to guide the inference process.

## 2. Statistical Basis for Data Integration

### 2.1. Pairwise Associations and Graphical Models

Typically, the basis for all omics analysis is formed by a large data matrix (or several, in case of multi-omics experiments): For gene expression data, for instance, the columns would represent individual genes (the variables) and the rows the different samples, each entry hence reflecting the expression of a certain gene in a specific sample.

Graphs are a common way of describing molecular interactions in such data, where nodes represent individual molecules and edges connecting the nodes represent their interactions. For instance, a graph might display proteins as nodes and represent protein-protein interactions as edges. Mathematically, nodes represent random variables (RVs) and integration methods seek to determine dependencies between RVs within or across omics types to infer interaction networks.

The simplest approach to construct correlation networks from multi-omics data is by applying pairwise association measures, such as linear regression, Pearson's Correlation Coefficient (PCC) or Spearman's Rank Correlation coefficient (SRCC), repeatedly on all pairs of RVs. Pairs with non-zero correlation coefficients will be connected by an edge in the graph (Figure 3B). For example, applying the PCC on the expression data of two genes which have been measured in multiple samples yields gene co-expression information: one gene is expressed when the other one is expressed or vice versa (similarly, one gene could be repressed while the other one is expressed). Furthermore, these measures are also used in quantitative-trait locus (QTL) based analyses to identify associations between e.g., SNPs and gene expression, i.e., to determine the genetic effect of sequence variation on a quantitative molecular trait. Alternatively, mutual information (MI) can be employed to detect non-linear relationships (Song et al., 2012). MI is used in several network inference tools (e.g., ARACNE, Margolin et al., 2006; Lachmann et al., 2016), but refined concepts of correlations like the biweight midcorrelation (Zhang and Horvath, 2005; Langfelder and Horvath, 2008) have been shown to outperform MI (Song et al., 2012).

**Figure 2:**
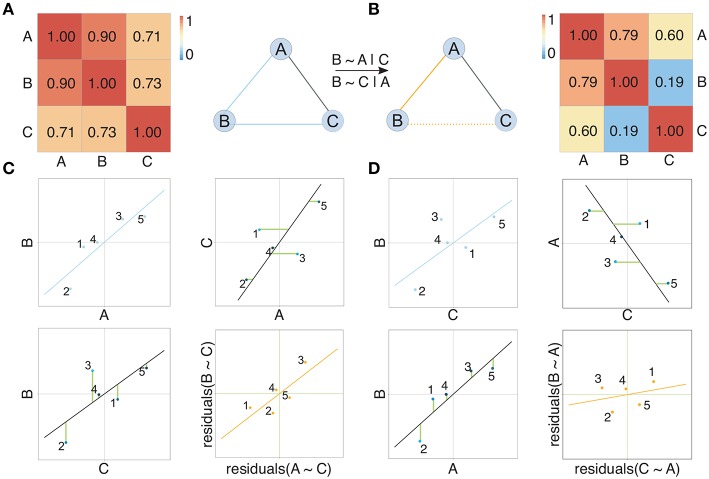
Illustration of the concept of partial correlation networks. Two networks show the dependency structure between random variables depicted as nodes. Solid edges in **(A)** represent high Pearson correlation coefficients between random variables, also shown in the corresponding correlation matrix. Solid edges in **(B)** represent non-zero partial correlation coefficients between random variables, also shown in the corresponding partial correlation matrix. Considering partial correlation compared to Pearson correlation removes the edge between B and C arising from the effect A exhibits on both B and C. Subfigure **(C)** compares correlation and partial correlation between A and B given C. Scatter plots show the original data (blue), the residuals (green lines) after regressing both A and B on C, and the relation between the residuals (orange). Here a clear linear relation between the residuals is observed, which is reflected in a non-zero partial correlation (represented by an edge) between A and B. Analogously, subfigure **(D)** compares correlation and partial correlation between B and C given A. Here no clear linear relation between the residuals is observed, which is reflected in a partial correlation between B and C that is not significantly different from zero. Consequently, there is no edge between B and C in the partial correlation graph.

**Figure 3:**
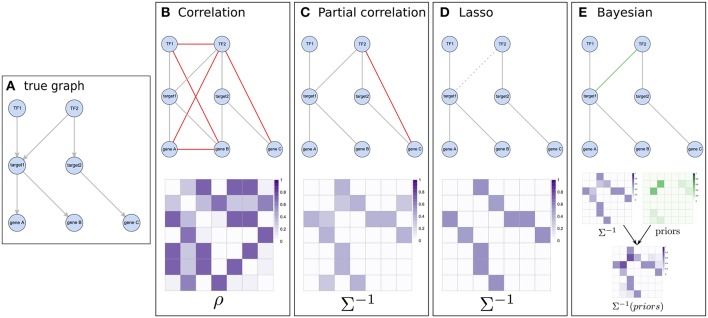
Illustration of the concept of different network inference methods. **(A)** represents a known pathway structure which should be recovered from functional data using the different approaches: two transcription factors influencing expression of two target genes which in turn affect the expression of other downstream genes. **(B,C)** show correlation based results and their estimated matrices (correlation and partial-correlation, respectively). While using Pearson correlation results in many indirect associations (shown in red), this is largely amended by using partial correlations. **(D)** The graphical lasso pushes weaker associations (e.g., between *TF1* and *gene C*) toward zero in the precision matrix and might do so even for real edges which have relatively low evidence in the data (like the edge between *TF2* and *target1*). **(E)** When considering prior information, weak associations still have a chance of getting selected if their respective prior (shown in green) supports them.

Pairwise approaches applied on omics data yield networks containing indirect associations due to their inability to distinguish direct and indirect effects (Schäfer and Strimmer, 2005). This leads to very dense networks (high number of edges) (Krumsiek et al., 2011) and hence limited interpretability. *Conditional dependencies* (partial correlations) associate two Gaussian variables while accounting for the effect of all other variables and thereby alleviate this problem: indirect dependencies between two variables originating from a direct dependency on a common source variable will no longer result in an additional connection between the two variables and only the direct interactions between the common source and each variable individually will be retained (Figures 2, 3C). As an example, consider the expression of two genes (node B and C in Figure 2) that are both regulated by the same transcription factor (node A in Figure 2). Regulation of a target gene by the transcription factor introduces a direct dependency between the expression of the transcription factor and the expression of the gene. If two genes are regulated by the same transcription factor, this dependence on a common source variable induces an indirect dependency between the two genes. This indirect dependency would introduce an edge in a pairwise correlation graph (Figure 2A), which would be removed when considering only conditional dependence measures such as partial correlation (Figures 2B,D). In contrast, direct dependencies such as the one between the transcription factor and its target (node A and B in Figure 2B) are preserved in the partial correlation network (Figure 2C). This idea is forming the basis of *graphical models*, known also as conditional dependence networks (Lauritzen, 1996; Meinshausen and Bühlmann, 2006; Friedman et al., 2008), where edges only represent the *conditional dependencies* between RVs.

In our case we mostly focus on *Gaussian Graphical Models* (GGMs) which assume normally distributed variables and have for instance been used in gene expression studies (Schäfer and Strimmer, 2005), in metabolomics (Krumsiek et al., 2011) and to discover novel interactions between genotypes and metabolites (Krumsiek et al., 2012). In GGMs, the network structure is given by the *precision matrix* Σ^−1^, the inverse of the covariance matrix Σ (Friedman et al., 2008) (see Box 1). In contrast to correlation networks, the edges reflect *partial correlations* (Figure 2) between RVs and correspond to the non-zero, off-diagonal entries of Σ^−1^. Methods seek to estimate either Σ^−1^ (e.g., GeneNet, Schäfer and Strimmer, 2005) or only its non-zero elements (e.g., Meinshausen and Bühlmann, 2006; Friedman et al., 2008).

### 2.2. Regularization and the Graphical LASSO

Inference methods working on genomic data typically suffer from the *n* < < *p* problem, which occurs when the number of samples is significantly smaller than the number of variables (a typical large expression experiment for example might comprise hundreds of samples and > 20, 000 genes). Specifically, if *n* < < *p*, fitting a statistical model is challenging: more variables than data points yield too many degrees of freedom and an underdetermined mathematical system, which ultimately poses a risk of overfitting the model to the measured data (Friedman et al., 2001). A way to handle this dimensionality burden is by using regularization (e.g., GeneNet Schäfer and Strimmer, 2005, see Box 1). While GeneNet uses a shrinkage procedure for estimating Σ^−1^, Meinshausen and Bühlmann apply LASSO (**L**east **A**bsolute **S**hrinkage and **S**election **O**perator) regression separately for each variable against all others to estimate the non-zero entries of Σ^−1^ under assumption of a sparse precision matrix (Meinshausen and Bühlmann, 2006). The underlying idea is to use *L*_1_ regularization (see Box 1) to constrain the total length of the estimated parameter vectors (variable coefficients, β = {β_1_, β_2_, …β_*p*_}) and simultaneously perform variable selection by implicitly pushing the least important parameters toward zero, thereby also circumventing the *n* < < *p* problem introduced above. This yields a precision matrix Σ^−1^ in which an entry σ_*ij*_ for two variables (*i, j*) is non-zero, if either β_*ij*_ (*i* regressed against *j*) or β_*ji*_ or both are non-zero. Ultimately, this procedure exhibits a similar effect as in the conditional dependence graphs mentioned above, with relatively more of the weaker dependencies getting removed (Figure 3D).

However, the above mentioned approach yields only an approximation of the underlying likelihood. To amend this, Friedman et al. present the *graphical LASSO* (*gLASSO*), which evaluates the penalized log-likelihood of the multivariate Gaussian distribution by using a block-wise gradient descent algorithm (Banerjee et al., 2007; Friedman et al., 2008) and other works improve upon this idea to achieve e.g., faster convergence (Hsieh et al., 2013).

An important task when using the above methods is to screen for optimal values of the *L*_1_ penalization parameter, ρ, to select the ideal graph. The selection of ρ can be done either via cross validation or by employing the **B**ayesian **I**nformation **C**riterion (BIC), where larger values of ρ encourage sparser graphs and vice versa.

Interestingly, the *gLASSO* also facilitates the inclusion of biological prior information [the LASSO *L*_1_ regularization can be interpreted as a Laplace prior on model coefficients (Friedman et al., 2001)]. By element-wise multiplication of a prior matrix *P* with Σ^−1^ in the *L*_1_-norm, where *P* = *p*×*p* and *p* is the number of variables, distinct weights can be assigned to encourage or discourage edges (Li and Jackson, 2015). Employing this possibility and in general utilizing prior information has the potential to retain edges which would otherwise falsely be removed (e.g., due to weak representation in the data) if the respective prior is strong enough (Figure 3E, see also section 2.4). Such prior knowledge, e.g., that a specific transcription factor is a known regulator of a gene (Figure 3E), can be extracted from independent databases such as the ones listed in Table 1.

### 2.3. Mixed Graphical Models for Multi-Omics Data Integration

Above mentioned methods assume Gaussian RVs and infer homogeneous networks, which is not always appropriate in multi-omics settings due to heterogeneity in the measured data. This is for example the case when integrating discrete genotype data with continuous DNA methylation data. This is taken into account by works building on the Meinshausen-Bühlmann approach to infer heterogeneous networks from multi-omics data using Mixed Graphical Models (MGMs) (Lee and Hastie, 2013) and identify edges by regressing each continuous or discrete variable against all others applying either (Gaussian) linear or (multiclass) logistic regression, respectively. This allows for example to directly integrate discrete genotype data of specific sequence variants with DNA methylation readouts and hence to uncover e.g., the genetic determinants of epigenetic marks. In contrast to Lee and Hastie who apply a single penalty parameter, Sedgewick et al. (2016) incorporate group-wise penalties, i.e., penalties for continuous-continuous, continuous-discrete and discrete-discrete edges, to account for different performances in edge prediction of linear and logistic regressions (Chen et al., 2014; Sedgewick et al., 2016). To achieve stable model selection under three distinct penalties, they propose a repeated subsampling procedure to determine the total instability of the model (StEPS, ***St****epwise* ***E****dge-specific* ***P****enalty* ***S****election*). The total instability reflects the average probability for edges to differ between two graphs estimated from subsamples of the data for all values of the penalty parameters. A threshold is applied on the screened regularization parameters such that the least amount of regularization is used to achieve a sparse, stable graph (Liu et al., 2010; Sedgewick et al., 2016).

An alternative to the *gLASSO* are tree based methods. A random forest model (Breiman, 2001; Hapfelmeier and Ulm, 2013) is built for each variable, using all other variables as predictors, and interactions are inferred by ranking variables according to their importance in explaining the selected variable similar to Meinshausen and Bühlmann (2006). Absence of any distributional assumptions (Huynh-Thu et al., 2010) and their ability to discover non-linear interactions and deal with large variable numbers harbors potential for use in multi-omics or in general mixed settings. Similar to the penalty parameter in the *gLASSO*, the number of edges to select from the individual models has to be optimized (Huynh-Thu et al., 2010; Fellinghauer et al., 2013), which can be achieved e.g. via Stability Selection (Meinshausen and Bühlmann, 2010) to control the number of false positive edges (Fellinghauer et al., 2013). Recent extensions further allow prior integration via weights included in the variable ranking (e.g., *iRafNet*, Petralia et al., 2015) or application to large single-cell datasets [e.g., *GRNBoost*, an extension to *GENIE3* (Huynh-Thu et al., 2010) used in the *SCENIC* workflow (Aibar et al., 2017)].

**Table 2:** List of network inference methods discussed in this review for which implementations are available.

**Method**	**Concept**	**Mixed data**	**Priors**	**Directed**	**References**
GeneNet	shrinkage/pcor	No	No	No	Schäfer and Strimmer, 2005
ARACNE(-AP)	Mutual information	No	No	No	Margolin et al., 2006; Lachmann et al., 2016
GENIE3	RF	Potentially^a^	No	No	Huynh-Thu et al., 2010
GRNBoost^b^	RF	Potentially^a^	No	No	Aibar et al., 2017
gLASSO	LASSO	No	No	No	Friedman et al., 2008
wgLasso	LASSO	No	No	No	Li and Jackson, 2015
pLasso	LASSO	No	No	No	Wang et al., 2013
iRafNet	RF	Yes	Yes	No	Petralia et al., 2015
GRaFo	RF/stability selection	Yes	No	No	Fellinghauer et al., 2013
causalMGM	RF/StEPS	Yes	No	Yes	Sedgewick et al., 2018
bdgraph	MCMC	yes	Yes	No	Mohammadi and Wit, 2015; Mohammadi et al., 2015

*Column concept describes the underlying statistical concept. Additional columns indicate applicability of methods to heterogeneous data types (mixed data) as well as possibility for prior incorporation (priors) or directed graph inference (directed). pcor, partial correlation; RF, random forest; StEPS, Stepwise Edge-specific Penalty Selection; MCMC, Markov Chain Monte Carlo*.

a *not specifically tarted to or evaluated with respect to this aspect*.

b *developed in single-cell context*.

### 2.4. Bayesian Treatment of Network Inference

A Bayesian approach for GGM estimation and prior incorporation is proposed by Mohammadi and Wit (Mohammadi and Wit, 2015). They estimate Σ^−1^ (the precision matrix, see Box 1) using a Markov-Chain-Monte-Carlo (MCMC) procedure. In brief, their approach samples from the large space of $2^{\frac{p*\left(p-1\right)}{2}}$ possible graph configurations (where p is the number of nodes/variables) and seeks the one best fitting the data and corresponding prior information. Their method *bdgraph* facilitates inclusion of edge-wise priors and extension to graphical copula models (Dobra and Lenkoski, 2011) allows integration of mixed data-types (Mohammadi et al., 2015). In contrast to MGMs, the copula is a semi-parametric approach which does not explicitly model different types of distributions but transfers non-normal variables to a Gaussian space before inferring the network.

While above approaches yield undirected associations and hence the direction of effect cannot be determined from the association, probabilistic Bayesian networks (BN) can be used to establish directed causal networks, e.g. indicating that expression of gene *B* changes as a result of an expression change of gene *A* (Zhu et al., 2004). BNs identify the best network by evaluating a likelihood together with prior information (Friedman et al., 2000) for numerous network structures (e.g., via MCMC sampling Zhu et al., 2007; Tasaki et al., 2015), which also allows integration of prior assumptions to guide the reconstruction (Zhu et al., 2007). However, BNs on their own cannot always reliably infer causality and additional evidence, e.g., from genetic data, are needed to infer edge directions, similar to Mendelian Randomization strategies (Zhu et al., 2004, 2007).

## 3. Heterogeneous Interactomes Using Asynchronous Integration

A simple way to integrate multiple omics data is to analyze pairs of data and integrate results in a step-wise fashion. Genotypes (e.g., SNPs) form the basis of inter-individual variation on the cellular level (Ritchie et al., 2015) and are therefore at the heart of many asynchronous methods. To decipher their mechanism of action, GWAS-SNPs are associated with quantitative molecular traits (quantitative trait loci, QTLs), using for example linear regression models to estimate their effects on mRNA expression (eQTLs), protein abundance (pQTL), DNA methylation (meQTLs), or metabolite levels (mQTLs). Of particular value for investigating regulatory interactions are *trans*-QTL hotspots: A SNP on one chromosome is associated with numerous traits such as gene expression levels of genes on different chromosomes. In order to explain the genome-wide changes a QTL hotspot variant induces in a cell it is necessary to understand the regulatory relationships giving rise to the observed *trans* associations.

For instance, *trans*-eQTLs can be used to analyze the consequences of disease associated SNPs on gene expression as was done by Võsa et al. (2018) (Figure 1). Here, the authors established *trans*-eQTLs at 3,853 unique SNPs associated to 6,298 unique genes in a large meta-analysis of whole blood data and describe the molecular effects of *trans* associated SNPs. Probing *trans*-eQTL loci for TFs encoded at the *trans*-acting locus and at the same time affected in gene expression locally by the variant along with ChIP-seq derived TF-DNA binding sites (TFBS) lead to an estimated 17.4% of *trans*-eQTLs whose effects could be explained by direct TF-target interactions. Similarly, for longrange-eQTL (same chromosome, distance ≥100 kb) they infer enhancer-promoter interactions and confirm physical DNA-DNA contacts using capture Hi-C data (Javierre et al., 2016). Following their approach, the authors were able to implicate for example circadian clock related genes with height as a complex trait, a hitherto unsuspected connection.

Bonder et al. interrogated GWAS-SNPs with regard to their impact on DNA methylation and gene expression in whole blood (Bonder et al., 2017). To analyze the influence of methylation on gene expression, they established associations between methylation and expression levels (expression quantitative trait methylation, eQTM) in addition to eQTLs and meQTLs (see Figure 1A). By integrating *trans*-meQTLs with eQTMs and TFBS from ChIP-seq data, they found disease loci to induce changes in gene expression networks via altered DNA binding of TFs (protein-DNA interactions) and DNA methylation changes (see Figure 1B). In their work, they extracted a novel gene network for a locus associated with ulcerative colitis (SNP *rs3774937*). They showed how this locus, residing in the first intron of the *NFKB1* gene, influences the expression of *NFKB1*, which in turn affects the methylation at distal CpG sites and further leads to a change in expression of genes close to those sites. Thereby, the authors established the molecular and regulatory interactions between *NFKB1*, methylation levels at the associated CpG sites and expression levels of the neighboring genes to generate hypotheses about molecular mechanisms underlying disease associations identified in GWAS (see Figure 1E).

Reference interactomes or *de-novo* gene co-expression networks allow a holistic view on the regulatory context of QTLs (Figure 1C). For example, after establishing *trans*-pQTLs for GWAS-SNPs, Suhre et al. (2017) connected *trans* associated traits by building PPI networks from a targeted protein expression assay (Gold et al., 2010) using GeneNet (Schäfer and Strimmer, 2005). They further joined pQTLs and their PPI network by adding genotype-protein edges for all identified pQTLs and contextualized their networks with disease information obtained from GWAS variants. Following this approach, the authors for instance gained novel insights into the molecular mechanisms involved in Alzheimer's disease (AD) by inferring a hitherto unknown link between a major AD risk variant (*rs4420638*) and splicing related proteins. They propose that a potential mediator of the effect of *rs4420638* on a splicing regulator (*SNRPF*) could be of pharmaceutical interest in order to decrease amyloid precursor protein levels, potentially improving understanding and treatment of AD (Suhre et al., 2017).

## 4. Synchronous Network Inference From Omics Data

While e.g., Bartel et al. (2015) inferred a transcriptome-metabolome network by step-wise application of the *SRCC* on all pairs of transcripts and metabolites, recovering known and unknown interactions, using all available data in a single integration step (synchronously) has the potential to boost inference performance by recognizing complementary regulatory information of other variables or omic layers (Petralia et al., 2015). To capture these effects and to make use of established knowledge (e.g., reference interactomes), graphical models often are preferred to pairwise approaches, specifically their extensions for heterogeneous network inference and prior inclusion. In the next three sections we will briefly present applications of synchronous inference methods, covering homogeneous and heterogeneous network inference, as well as prior based inference approaches.

### 4.1. Homogeneous Network Reconstruction

Krumsiek et al. (2011) used a large metabolite dataset on which they applied a GGM based approach to infer *de-novo* metabolite reaction networks (see Figure 1C). Although only in a single-omics setting, they were able to demonstrate the added benefit of using network based inference as compared to pairwise approaches: by comparing their inferred network to known metabolic reactions as a reference interactome (e.g., from KEGG, see Table 1), they were able to propose additional associations (Figure 1D) between lipid metabolites which had so far only indirectly been associated in the reference.

With the advance of single-cell experiments, recent studies seek to make use of their favorable statistical properties (e.g., large sample sizes) in association analyses. Specifically, single-cell protocols have been proposed to assess multiple regulatory layers in individual cells (e.g., scNMT-seq Clark et al., 2018, sciCAR Cao et al., 2018, or scCAT-seq Liu et al., 2019). However, these data come with their own challenges such as dropout effects, large number of missing values and technical variation, which have to be overcome to use them to their full potential (Colomé-Tatché and Theis, 2018).

Aibar et al. (2017) for example proposed the single-cell regulatory network inference and clustering (*SCENIC*) workflow to map gene regulatory networks in single-cell data and identify stable cell states of individual cells based on common regulatory subnetworks. The authors seek to overcome general limitations of single-cell data by integrating *cis*-regulatory sequence analysis with single-cell gene expression data and provide an extension to *GENIE3* (Huynh-Thu et al., 2010), *GRNBoost*, which scales favorably with respect to computation time in large (single-cell) datasets.

In another study, Pliner et al. used a *gLASSO* based approach (*CICERO*) to identify co-accessibility regions from single-cell ATAC-seq data (Pliner et al., 2018). Those regions represent distal regulatory elements that interact with DNA regulatory elements at the promoters of the respective target genes. Comparison of their results with physical interactions measured using promoter-capture Hi-C (Cairns et al., 2016) showed a strong overlap, suggesting physical interactions between the co-accessibility regions detected through their network inference approach. Similar regulatory inference can be performed based on scCAT-seq as the authors demonstrated by example of inferring regulatory relationships between accessible chromatin regions and expression of putative target genes (Liu et al., 2019).

### 4.2. Inference of Heterogeneous Networks

Saha et al. (2017) used GTEx gene expression data to infer transcriptome-wide (TWNs) and tissue-specific (TSN) networks using GGMs (Hsieh et al., 2013). Using RNA-seq data, the authors define a heterogeneous network containing total expression (TE) or isoform ratio (IR) nodes, enabling the investigation of splicing control mechanisms e.g., by observing TE-IR edges indicating potential splicing regulators. They further constructed different *L*_1_ penalties for distinct edge types (TE-TE, TE-IR, or IR-IR) to encode prior assumptions for their occurrence (similar to Sedgewick et al., 2016). With their strategy, the authors were able to recover known (e.g., *RBM14, PPP1R10*) and propose novel (e.g., *TMEM160*) splicing regulators across different tissues as well as pinpoint tissue-specific regulators such as *TTC36* in breast-mammary tissue which could be essential to unravel disease related regulatory mechanisms. For example, they identified *MAGHO* and *MAB21L1* as hub genes (i.e., strongly connected genes) in brain-caudate and artery-aorta specific TSNs, respectively. Both genes have been found to play an important role in tissue-specific transcription regulation and are known to be crucial for the development of their TSN's respective organs.

Due to the relatively novel idea of using multi-omics data for heterogeneous network inference, MGM applications are mostly limited to proof of concept studies with simulated data (Lee and Hastie, 2013; Haslbeck and Waldorp, 2016). Although MGMs have been shown to perform well, further investigations are needed to demonstrate their usefulness in real-world contexts.

An interesting line of work in this direction is the inclusion of phenotype information. The tree based method proposed in Fellinghauer et al. (2013) (graphical random forests, *GRaFo*), for example, was used in a multi-omics study by Zierer et al. to evaluate age related disease comorbidities (Zierer et al., 2016) and their dependencies on molecular traits from transcriptomics, metabolomics, epigenomics, and glycomics. Here, the authors established a heterogeneous network and identified for example urate as a key factor linking metabolic syndrome phenotypes to renal function and body composition.

Another line of work with respect to the application of graphical models in disease contexts is given by Mohammadi et al. (2015). In their study of Dupuytren disease (a disease affecting finger contractures), the authors apply their extended *bdgraph* approach to model indicators and severity of the disease together with 13 different potential risk factors. Although Mohammadi and colleagues did not use omics data in their case study, they demonstrate the possibilities of heterogeneous network inference to elucidate disease pathogenesis: They affirmed a possible genetic risk for the disease as well as identify key phenotypic factors, such as age and alcohol consumption, which have a direct impact on the severity of Dupuytren disease. They further found that the severity of the disease is correlated for individual fingers and proposed to perform surgical measures simultaneously for both the ring and the middle finger as an improved therapy as compared to treating them independently.

### 4.3. Leveraging Biological Prior Knowledge for Network Reconstruction

Given the broad availability of reference interactomes, a significant amount of work focused on using them to improve network reconstruction. These works guide network inference by setting weights on specific edges, e.g. generated by combining reference interactomes with omics data (Figures 1B,D).

Wang et al. (*pLasso*) and Li and Jackson (*wgLasso*) use reference interactomes in an adjusted LASSO and *gLASSO* context (Wang et al., 2013; Li and Jackson, 2015). Wang et al. (2013) extracted interaction networks from KEGG and the Pathway Commons database (see Table 1) to define distinct penalties for *prior* and *non-prior edges*, i.e., nodes linked or not linked in the reference network, respectively. Li and Jackson (2015) showed that using priors in *wgLasso* outperforms the regular *gLASSO* on simulated data as well as real-world gene expression data from *Arabidopsis thaliana* compared to a reference of annotated gene pathways (Lee et al., 2010) in terms of the Matthew's Correlation Coefficient (MCC).

MGM based methodologies have also been extended to incorporate prior information. For example, Manatakis et al. (2018) proposed *prior incorporation Mixed Graphical Models* (*piMGM*), an extension to *CausalMGM* (Sedgewick et al., 2016, 2018). *piMGM* independently applies *CausalMGM* for a set of regularization parameters on a random partition of samples and assembles the final graph by aggregating all generated models. This method encourages specific network edges via incorporation of pathway knowledge similar to *pLasso* and *wgLasso*. They evaluated their approach via breast cancer subtype prediction in TCGA RNA-seq and cancer subtype data with priors derived from KEGG pathways and were able to recover known pathways (e.g., the *Notch* signaling pathway) as well as determine the parts of the pathways most important to breast cancer subtyping. In addition, they affirmed a possible role of other pathways, such as the insulin signaling pathway or T cell receptor signaling, as an important part in determining cancer subtypes.

Petralia et al. (2015) applied *iRafNet* on test data from two “Dialogue for Reverse Engineering Assessments and Methods” (DREAM4/5) challenges (Greenfield et al., 2010; Marbach et al., 2012) and used prior information obtained from time-series gene expression and knockout data in addition to PPIs to infer regulatory networks. They demonstrate improved performance as compared to *GENIE3* (which does not utilize prior information) in terms of *Area Under the receiver operator characteristic Curve* (AUC) and *Area Under the Precision Recall Curve* (AUPRC) (Petralia et al., 2015). However, as for *GENIE3*, this approach has not yet been applied to mixed data types.

Finally, Zhu et al. (2008) derived directed yeast regulatory networks with a BN approach. Using a modified MCMC strategy (Friedman et al., 2000; Zhu et al., 2004), they inferred a causal consensus network from 1,000 sampled networks (edges present in ≥30% of the networks) and determined edge directions by including PPI, TFBS, and eQTL information as priors. They used gene knockout data to demonstrate the predictive power of their network in determining the downstream effects of systematic changes in the biological system. Their study lead to the prediction of novel gene interactions, complementing existing yeast PPI databases, as well as pinpointing novel causal drivers of yeast eQTL hotspots. Strikingly, they were able to confirm their computationally derived interaction predictions by using previous experimental findings and hence showcase the predictive power of their approach. For instance, they identified *AMN1*, a gene implicated in yeast daughter cell separation, as a causal transcriptional regulator based on one of their eQTL hotspots, a discovery made in experimental screens by Yvert et al. (2003).

## 5. Conclusion

To understand disease causing molecular processes, systems biology studies seek to establish molecular interaction networks or *interactomes*. Numerous methods have been developed for contextualizing reference interactomes from large databases and to pinpoint interactions important in disease with the help of multi-omics data.

While many studies perform step-wise data integration, relatively few studies follow synchronous integration strategies for constructing homogeneous or heterogeneous networks, which could exploit omics data to their full potential and therefore are representing promising tools to unravel complex cellular processes.

Methods previously used for constructing homogeneous networks (e.g., *GENIE3*, Huynh-Thu et al., 2010, for gene expression data) could be applied to multi-omics data to infer heterogeneous networks, however, additional evaluation and benchmarks are required. Yet, as the top performer in two DREAM challenges (DREAM4/5, Greenfield et al., 2010; Marbach et al., 2012), *GENIE3* and, more generally, tree based methods represent a promising basis for multi-omics network inference.

Most recent methods implement variations of the *graphical LASSO* to predict conditional dependence networks from experimental data (Meinshausen and Bühlmann, 2006; Friedman et al., 2008). Additionally, methods like *wgLasso, piMGM, iRafNet*, and *bdgraph* can utilize prior knowledge to guide the inference process. With these methods, large public databases containing massive multi-omics data represent important assets for network inference and to contextualize reference interactomes.

To date only few studies make use of these independent data (e.g., Li and Jackson, 2015; Sedgewick et al., 2018) and current methods such as *iRafNet* and *piMGM*) need to be adjusted and applied to new biological contexts to make full use of their potential. An interesting challenge with respect to including prior information in computational models, for example, is to make the plethora of available biological data accessible to such methods, i.e., to create data driven priors not only relying on available PPI databases but making use of further data such as available chromatin conformation data, DNA accessibility or other biological knowledge.

Moreover, novel experimental protocols such as scNMT-seq (single-cell nucleosome, methylation and transcription sequencing) (Clark et al., 2018), sciCAR (Cao et al., 2018), or scCAT-seq (Liu et al., 2019) allow for simultaneously probing multiple molecular layers in hundreds of individual cells. Such new methods, and in general the development of single-cell techniques, pave exciting new avenues for the analysis of cell-type specific networks and initial studies show promising results (Moignard et al., 2015; Aibar et al., 2017; Pliner et al., 2018). Nevertheless, methods have to be further adapted to be able to cope with single-cell contexts, e.g., to take into account dropout effects and differing noise properties (Colomé-Tatché and Theis, 2018).

In addition to the methods discussed above, protocols to directly measure interacting molecules from biological samples are steadily improving. More reliable experimental protocols to e.g., measure protein-metabolite interactions (Piazza et al., 2018) or to establish genome-wide protein-RNA interactions (Van Nostrand et al., 2016) could improve reference interactome quality, in turn alleviating reconstruction of context-specific interactomes.

Finally, other strategies for network based integration of molecular data such as methods implementing network diffusion (e.g., Dimitrakopoulos et al., 2018) or network embedding (e.g., Perozzi et al., 2017) could be used to complement network inference efforts and have in fact been shown to improve the predictive performance of biomedical networks (Su et al., 2018). Indeed, some methods (e.g., by Kuchaiev et al., 2009) can even be used to refine (de-noise) reference interactomes and predict novel interactions (not part of this review). However, most such methods rely heavily on established molecular networks, making initial network creation a crucial step for their successful application.

## Author Contributions

JH, FT, and MH wrote the manuscript.

### Conflict of Interest Statement

The authors declare that the research was conducted in the absence of any commercial or financial relationships that could be construed as a potential conflict of interest.
